# Experiences of renal healthcare practitioners during the COVID-19 pandemic: a multi-methods approach

**DOI:** 10.1186/s12882-021-02500-0

**Published:** 2021-09-07

**Authors:** Clare Mc Keaveney, Joanne Reid, Claire Carswell, Ann Bonner, Ilaria de Barbieri, William Johnston, Alexander P. Maxwell, Julien O’Riordan, Veronica Strini, Ian Walsh, Helen Noble

**Affiliations:** 1 School of Nursing and Midwifery, Queens University Belfast, Belfast, UK; 2 Department of Health Sciences, University of York, York, UK; 3 Griffith University, Brisbane, Australia; 4 Padua University Hospital, Padova, Italy; 5 Kidney Care, Alton, UK; 6 Northern Ireland Kidney Patient Association, Belfast, UK; 7 Regional Nephrology Unit, Belfast City Hospital, Belfast, UK; 8 Centre for Public Health, Queens University Belfast, Belfast, UK; 9 Galway Hospice Foundation, Galway, Ireland; 10 National University of Ireland, Galway, Ireland; 11 School of Medicine, Dentistry and Biomedical Sciences, Queens University Belfast, Belfast, UK

**Keywords:** Renal, Burnout, COVID-19, Healthcare Practitioners, Multi-methods, Well-being

## Abstract

**Background:**

Globally, renal healthcare practitioners provide intensive and protracted support to a highly complex multi-morbid patient population however knowledge about the impact of COVID-19 on these practitioners is extremely limited.

**Objective:**

This study aimed to explore the experiences of COVID-19 with renal healthcare practitioners during the first global lockdown between June 2020 and September 2020.

**Methods:**

A multi-methods approach was carried out including a quantitative survey and qualitative interviews. This was a multinational study of renal healthcare practitioners from 29 countries. *Quantitative*: A self-designed survey on COVID-19 experiences and standardised questionnaires (General Health Questionnaire-12; Maslach Burnout Inventory). Descriptive statistics were generated for numerical data. *Qualitative*: Online semi-structured interviews were conducted. Data was subjected to thematic analysis. Renal healthcare practitioners (*n* = 251) completed an online survey. Thirteen renal healthcare practitioners took part in semi-structured interviews (12 nurses and 1 dietician).

**Results:**

The majority of participants surveyed were female (86.9 %; *n* = 218), nurses (86.9 %; *n* = 218) with an average 21.5 (SD = 11.1) years’ experience since professional qualification, and 16.3 years (SD = 9.3) working in renal healthcare. Survey responses indicated a level of preparedness, training and satisfactory personal protective equipment during the pandemic however approximately 40.3 % experienced fear about attending work, and 49.8 % experienced mental health distress. The highest prevalence of burnout was emotional exhaustion (35.9 %). Three themes emerged from the qualitative analysis highlighting the holistic complexities in managing renal healthcare, a neglected specialist workforce, and the need for appropriate support at work during a pandemic.

**Conclusions:**

Results have highlighted the psychological impact, in terms of emotional exhaustion and mental health distress in our sample of renal healthcare practitioners. As the pandemic has continued, it is important to consider the long-term impact on an already stretched workforce including the risk of developing mental health disorders. Future research and interventions are required to understand and improve the provision of psychological support for specialist medical and nursing personnel.

**Supplementary Information:**

The online version contains supplementary material available at 10.1186/s12882-021-02500-0.

## Background

The coronavirus disease 2019 (COVID-19) outbreak was designated a public health emergency of international concern [1]. Since this initial outbreak the virus has spread to every continent and has been declared a pandemic by the World Health Organisation, with approximately 170 million confirmed cases, 3.5 million deaths worldwide [2]. The highest mortality rates have been in people over the age of 60 and those who are immunocompromised or have underlying health conditions [3]. The outbreak has pressured healthcare systems across the globe [4]. While some countries have tried to adapt, for example by recruiting additional healthcare workers, building new facilities and mass producing necessary medical equipment [5], the significant spread of the virus has meant additional broader societal lockdown measures have been implemented to manage the demands on healthcare services and to reduce the escalating mortality rates [6].

Healthcare practitioners (HCPs) have been at the forefront in managing this global pandemic, providing treatment and diagnostic testing to patients who have suspected COVID-19 [7]. Studies investigating the experiences of HCPs during the COVID-19 pandemic have been predominantly quantitative [8] showing heightened stress, anxiety, and depressive symptoms as a result of working during the COVID-19 pandemic [9]. However, there remains a lack of ‘*qualitative approaches to capture the real-world experiences of frontline [staff]’* ([10] p.21). Additionally, evidence has predominantly focused on anaesthesiology, radiology, and immunology HCPs [11, 12]. Other specialties such as nephrology during the initial outbreak has been neglected [4]. This is surprising given COVID-19 related acute kidney injury [13, 14] has increased the demand for kidney services, leading to concerns about renal HCPs as well as shortages of dialysis equipment, and the potential need to ration treatment [15].

Nephrology is a complex discipline including kidney disease, dialysis and transplantation [16]. Renal HCPs provide intensive and protracted support to a highly complex multi-morbid patient population. Many patients have reduced physical functional abilities, diminished health-related quality of life, increased disease comorbidities and high mortality rates [17] and the impact of COVID-19 has brought additional burdens. Patients with kidney failure (previously known as end-stage kidney disease [18]) are significantly more at risk of severe COVID-19 infection and particularly those patients receiving kidney replacement therapies such as haemodialysis or transplantation [19, 20]. Haemodialysis units are high-risk areas as enclosed, clinical spaces placing patients and HCPs at greater risk of disease transmission. Renal HCPs face the challenge of providing high quality care to patients whilst utilising expanded infection control measures to reduce the risk of exposing high-risk patients to the virus.

These significant stressors are impacting heavily on staff, so the mental health and well-being of renal HCPs is an urgent priority to limit burnout and to sustain their ability to engage effectively in clinical work [4]. Knowledge about the impact of COVID-19 on renal HCPs is absent. This study aims to conduct a multi-method approach to investigate the impact of COVID-19 on renal HCPs working in multiple countries.

## Methods

### Study design

A multi-method study using an online survey and semi-structured interviews. Approval was received from the Faculty of Medicine, Health and Life Sciences ethical committee (MHLS 20_59) within the host institution.

### Study participants

 Participants were recruited in collaboration with the European Dialysis and Transplant Nurses Association/European Renal Care Association (EDTNA/ERCA) and associated international renal networks via social media. Data was collected online via a web-based survey tool (Qualtrics®). Renal HCPs were recruited during the first global lockdown between June 2020 and September 2020 using non-probability convenience sampling. The EDTNA/ERCA (*n* = 850) and the Renal Society of Australasia (RSA; *n* = 1400) sent an invitation email to all members. The EDTNA/ERCA is a European network established in 1971 to address the educational needs of nurses and other healthcare practitioners caring for patients who have chronic kidney disease. The RSA has similar goals to the EDTNA/ERCA.

### Data collection

#### Quantitative

Demographic information was collected (e.g. age, nationality, healthcare discipline, years since qualified). Standardised questionnaires were completed (Maslach Burnout Inventory [21] and the General Health Questionnaire [22]). Both questionnaires have been administered and validated within a range of HCPs including nephrology [17, 23]. Burnout: The Maslach Burnout Inventory (MBI) was used to assess levels of staff burnout. The inventory is a 22-item measure that assesses the frequency of burnout within three domains: emotional exhaustion, depersonalisation and personal accomplishment using the most commonly used cut-offs: high emotional exhaustion (≥ 27), high depersonalisation (≥ 10) and low personal accomplishment (≤ 33; [24]). Mental health distress: The General Health Questionnaire–12 (GHQ-12) measures twelve symptoms of psychological distress. GHQ-12 is reported using the Likert-scale (e.g., 0-1-2-3), higher scores indicating greater psychological distress (range 0–36; [25]) and as ‘cases’ using the bi-modal scale (e.g., 0-0-1-1) with ≥3 indicating psychological distress [26]. *Qualitative*: A broad topic guide was devised (see supplementary file 1) involving open-ended questions and probes to ensure vital information was not lost [27]. Questions were based on feelings, experiences and knowledge of participants working in nephrology during COVID-19 pandemic. All interviews were conducted online in English, digitally recorded, and transcribed verbatim for analysis.

### Data analysis

#### Quantitative

Quantitative: Online survey data were exported into SPSS 26. Descriptive statistics (frequencies, percentages, means, standard deviation [SD] and 95 % confidence intervals [CI]) were generated for each response. Qualitative: Analysis of the free text questions and open-ended questions was carried out using a thematic analysis framework developed by Miles and Huberman [28]. This framework consists of three concurrent flows of activity: data reduction, data display and conclusion drawing/verification. Data reduction refers to the process of selecting, focusing, simplifying and transforming the data from transcripts. In this study, data reduction was completed by transcribing the recorded semi-structured interviews verbatim, reading the transcripts in order to get an understanding of the data and undertaking initial coding. To ensure rigour, coding and grouping similar codes (i.e. simplification) and then transforming into themes was completed by the lead author (CMcK). Independent coding and verification of themes was completed within the research team (JR, HN). To facilitate data organisation and categorisation NVivo 12 software was used.

## Results

Between June 2020 and September 2020 an online survey was completed by 251 renal HCPs. Thirteen renal HCPs completed semi-structured interviews (12 nurses and 1 dietician).

### Survey responses

**Fig. 1:**
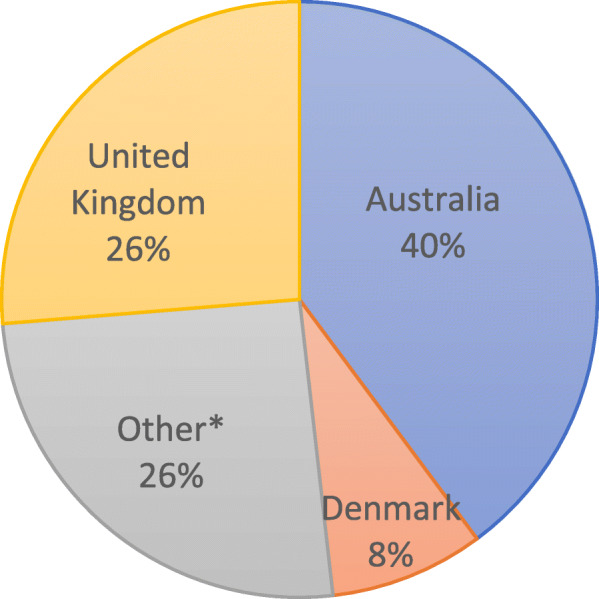
Graph of global breakdown of respondents. *Canada, Croatia, Cyprus, Finland, France, Germany, Greece, Ireland, Israel, Italy, Lebanon, Macedonia, Netherlands, New Zealand, Norway, Portugal, Romania, Saudi Arabia, Serbia, Slovenia, South Africa, Spain, Sweden, Turkey, United Arab Emirates, United States

Table 1 provides demographic information. The majority of participants were female (86.9 %; *n* = 218), aged between 45 and 54 (37.1 %; *n* = 93), in the nursing profession (86.9 %; *n* = 218), married (73.7 %; *n* = 185) and a culminative 59.4 % did have caring responsibilities (37.5 %, children; 2.4 %, other relative; 5.2 % other; 10.8 % parent; 3.5 % relative with medical condition). The average number of years’ experience since initial professional qualification was 21.5 (SD = 11.1) years and participants had an average of 16.3 (SD = 9.3) years working in nephrology. Participants from 29 countries completed the survey (Fig. 1) with the majority of respondents from Australia (*n* = 100), the United Kingdom (*n* = 66) and Denmark (*n* = 21).

**Table 1:** Demographic information of respondents

Demographics	Frequency (%)
**Gender**	
Female	218 (86.9%)
**Age**	
25-34 years	31 (12.3%)
35-44 years	64 (25.5%)
45-54 years	93 (37.1%)
55-64 years	61 (24.3%)
65+years	2 (0.8%)
**Profession**	
Dietician	6 (2.4%)
Medical Practitioner	11 (4.4.%)
Nurse	218 (86.9%)
Pharmacist	2 (0.8%)
Other	14 (5.5%)
**Marital status**	
Single	42 (16.7%)
Married/Co-habiting	185 (73.7%)
Widow	4 (1.6%)
Divorced/separated	20 (8.0%)
**Caring responsibilities**	
Children <18 years	94 (37.5%)
Other relative	6 (2.4%)
None	102 (40.6%)
Other	13 (5.2%)
Parent	27 (10.8%)
Relative with medical condition	9 (3.5%)
**Experience**	
Years since qualification	21.5 +/-11.1 (mean/SD)
Years in the renal speciality	16.3 +/- 9.3 (mean/SD)

Table 2 provides the frequencies of reported experiences of COVID-19. Less than half of the respondents were tested for COVID-19 (tested but negative, 39.0 %; (*n* = 98); yes, and recovering, 2.0 % (*n* = 5)) however most were working in a facility (77.3 %; *n* = 194) and provided direct care for COVID-19 positive patients (55.8 %; *n* = 140). The majority of respondents had not faced circumstances where they had to self-isolate due to COVID-19 (68.5 %; *n* = 172). Of those who did isolate due to COVID-19 (31.5 %; *n* = 79), only 35.4% (*n* = 28) had to isolate away from their own home. Just under a quarter of respondents cared for a patient who died of COVID-19 (22.3 %; *n* = 56) and sixteen (6.4 %) respondents were personally bereaved by COVID-19. Generally, respondents felt confident that they understood the guidelines that had been set by their employer regarding COVID-19 (60.1 %; *n* = 151), with the majority feeling ‘somewhat prepared’ to provide care for a patient with known or suspected COVID-19 (49.0 %; *n* = 123), and felt adequately trained to work during the COVID-19 pandemic (63.3 %; *n* = 159). It was reported that most facilities had a plan in place to care for those with known or suspected COVID-19 (94.4 %; *n* = 237), to screen and treat patients who came into the facility to make sure that patients with possible COVID-19 were isolated (87.2 %; *n* = 219). However nearly one-third of respondents felt Personal Protective Equipment (PPE) was not satisfactory during the pandemic (22.3 % (*n* = 56) reporting PPE was ‘not sufficient’ and 10.4 % (*n* = 26) were ‘unsure’). Subsequently, 40.3 % (*n* = 101) of all respondents felt afraid to come to work during the COVID-19 pandemic and many reported mental health and well-being support would be helpful whilst working as a renal HCP during the pandemic (76.9 %; *n* = 193).

**Table 2:** Reported survey responses for COVID-19 related questions

Survey item	Frequency (%)
**Ever been tested for COVID-19?**	
Tested but negative	98 (39.0%)
No	138 (55.0%)
Not formally but suspected	10 (4.0%)
Yes, and recovering	5 (2.0%)
**How many times tested**? (Mean (range))	1 (1-10)
**Worked at a facility with COVID-19 patients?**	
No	44 (17.5%)
Yes	194 (77.3%)
Unsure	13 (5.2%)
**Provided direct care to COVID-19 patients?**	
No	104 (41.4%)
Yes	140 (55.8%)
Unsure	7 (2.8%)
**Had to isolate due to COVID-19?**	
Yes	79 (31.5%)
No	172 (68.5%)
**Had to isolate away from home?**	
Yes	28 (35.4%)
No	51 (64.6%)
**Cared for patients who have died of COVID-19?**	
Yes	56 (22.3%)
No	179 (71.3%)
Unsure	16 (6.4%)
**Personally bereaved by COVID-19?**	
Yes	16 (6.4%)
No	235 (93.6%)
**Do you understand the guidelines that have been set by your employer regarding COVID-19?**	
No, I have questions	11 (4.4%)
Somewhat	7 (2.8%)
Yes, confidently understanding	151 (60.1%)
Yes, I think so	82 (32.7%)
**How prepared do you feel to provide care for a patient with a known or suspected COVID-19?**	
Somewhat prepared	123 (49.0%)
Somewhat unprepared	9 (3.6%)
Unsure	16 (6.4%)
Very prepared	96 (38.2%)
Very unprepared	7 (2.8%)
**Have you had sufficient PPE during the COVID-19 pandemic?**	
No	56 (22.3%)
Unsure	26 (10.4%)
Yes	169 (67.3%)
**Do you feel you received adequate training to work during the COVID-19 pandemic?**	
No	62 (24.7%)
Unsure	30 (12.0%)
Yes	159 (63.3%)
**Does your facility have a plan in place to care for those with known or suspected COVID-19?**	
No	5 (2.0%)
Unsure	9 (3.6%)
Yes	237 (94.4%)
**Is there a place in your facility to screen and treat patients who come into the facility to make sure that patients with possible COVID-19 are isolated?**	
No	21 (8.4%)
Unsure	11 (4.4%)
Yes	219 (87.2%)
**Have you ever felt afraid to come to work due to COVID-19 pandemic?**	
Yes	101 (40.3%)
No	150 (59.7%)
**Would mental health and well-being support be helpful to you whilst working as a renal Health Care Practitioner during the COVID-19 outbreak?**	
Yes	193 (76.9%)
No	58 (23.1%)

#### Levels of burnout and mental-health distress

Maslach Burnout Inventory provided burnout scores for respondents (*n* = 251). Overall mean scores showed moderate emotional exhaustion (m = 21.5, SD = 11.9), low depersonalisation (m = 5.3, SD = 4.7) and moderate personal accomplishment (m = 38.6, SD = 6.4; see supplementary file 2).

In addition, 90 participants (35.9 %) had severe levels of emotional exhaustion; 42 (16.7 %) had severe levels of depersonalisation; and 53 (21.1 %) had low levels of personal accomplishment (Table 3).

**Table 3:** Maslach Burnout Inventory (MBI): Mean scores and high-risk cases for HCPs

		Domains		
		Emotional exhaustion >27	Depersonalisation >10	Lack of personal accomplishment <33
Renal HCPs	Mean (SD)	21.5 (11.9)	5.3 (4.7)	38.8 (6.4)
	Cases %	35.9%	16.7%	21.1%

The General Health Questionnaire mean mental health score was 14.0 (CI = 13.2–14.8). Mental health distress (scores ≥3) was found in almost half of respondents (*n* = 125, 49.8 %; Table 4).

**Table 4:** General Health Questionnaire 12 (GHQ-12)

**GHQ-12 mean total score (CI 95%)** (using Likert method)	14.0 (13.2 – 14.8)
**Proportion with significant level of mental distress** (using bi-modal method)	49.8%

### Qualitative findings

Twelve renal nurses and one renal dietician completed the semi-structured interview from nine countries; Australia, Denmark, Greece, Italy, Lebanon, Lithuania, Saudi Arabia, Slovenia, and the United Kingdom. Data saturation was achieved with the 13th participant. Themes identified were: managing COVID-19 within a renal clinical environment (e.g., failing facilities, shortage of staff, information overload), the holistic impact of COVID-19 on staff (e.g., anxiety, psychological and physical exhaustion) and safeguarding HCPs working in nephrology (e.g., building resilience, new organisational pathways).

#### Managing COVID-19 within a renal setting

In-hospital life-sustaining treatments and appointments continued throughout the COVID-19 pandemic for patients receiving haemodialysis and, in some countries, transplantation, however, participants conveyed the challenges in adapting the clinical environment to the mounting COVID-19 pandemic,

"… [heads of dept] didn’t consider that we are a specialised [unit] and that our patients could get COVID-19 as well…just kind of find out along the way” and “Whilst the hospital has effective screening and isolating of COVID suspected patients, satellite dialysis patients are not as well catered for. The unit is old and crowded with only 1 single room. It is very difficult to truly isolate a patient…"

Staff working during the pandemic reported being on *‘high alert’* whilst trying to maintain a calm working environment in the face of significant change and uncertainty. This included managing exorbitant information from mass media. Guidelines from national health organisations and professional bodies on COVID-19 were reported as '*frequent and fast-changing*' and sometimes not providing enough disease-specific information. Over time HCPs had no option but to use this information flexibly to meet the requirements of these specialist units,

"When everything begins, we received a lot of recommendations from government and from our quality department. And of course, the recommendation was very wide internally. And we looked what is really appropriate for us and really [what would] fit…"

Recommendations and controls regarding PPE led to additional challenges in the renal unit. HCPs confirmed PPE was not always available including access to face masks. This led some departments igniting monitoring of said PPE, also creating additional workloads. Staff also described dehydration whilst on shift due to a reluctance to drink water in-between breaks as this would require a change of PPE. A key concern for HCPs involved improving patient compliance with COVID-19 infection control regulations including wearing masks. In some clinics, all eating and drinking was removed for patients to prevent masks from being removed,

“Wearing PPE to greet all patients, [it is] a barrier to normalcy and hot to wear. The PPE is exhausting and the emotional impact of caring for people who are fed up from shielding mixed with the fear of working with positive cases and contracting COVID-19 is exhausting. There just seems to be no let-up”.

Participants acknowledged COVID-19 restrictions also created barriers supporting patients during the pandemic. For example, masks and distancing rules created communication difficulties. This led to unmet emotional and psychological needs of patients, growing patient fears about attending hospital and missed patient appointments. Despite having suspected cases of COVID-19, patients with kidney failure requiring maintenance haemodialysis, had to be encouraged to attend for treatment causing considerable worry and concern for HCPs,

“…Our patients had no option to stay away. Even if sick they had to come. In the first week of lockdown, we had inadequate PPE, and the rules changed hour by hour which was incredibly stressful. I went home wondering if I had done things wrong…”.

Long-standing challenges such as staff shortages and staff sickness were further amplified within nephrology departments during the pandemic. In addition, as experts in specialist nephrology, many felt undervalued and expressed concerns about the safe delivery of care in unfamiliar clinical care environments, when placed under redeployment during the pandemic care. This stemmed from the evolving reconfiguration and reduction of services; collectively, these experiences placed additional strains on a specialist and smaller workforce,

"…you are asking the same people time and time again to keep going...so you are asking the same people to continually do one surge, do a big catch up, do a second surge and do an even bigger catch up. So that is difficult…"

#### Holistic impact of COVID-19 on staff

Many HCPs described emotional, psychological, and physical exhaustion while working during the pandemic. Inadequate facilities, limited resources and increased risk of COVID-19 infection, created a constant sense of fear in HCPs. In the event of being ‘inundated’ with patients with COVID-19, HCPs were concerned about being exposed, spreading or dying from the infection as well,

“I work with a great bunch of people the hardest thing would be if one of our patients or staff passed away from this bloody disease”.

Concerns about contracting the virus also led HCPs to avoid their own family and friends placing an additional strain on important emotional and social relationships outside of work. Many HCPs expressed a significant loss of support and feelings of loneliness while working during the pandemic. The lack of connectedness led to HCPs feeling more socially isolated as well as more depressed and anxious,

“For me, the hardest part is being unable to return to my family, because if I do I will not be able…to return to work”.

Time off from work due to illness or loss of childcare carried feelings of guilt and shame; guilt over knowing colleagues would be required to cover shifts, and shame when personally diagnosed with, or suspected of having, COVID-19. HCPs were also the brunt of patient frustration, reporting abuse from patients during the pandemic. This led to feelings of being overwhelmed and some had doubts about continuing to nurse in the future,

“But partly it was challenging the whole concept of nurses as saints and saying well no, actually…it worries me this whole putting a halo on the healthcare workers at the moment, because there might be this expectation that you’ll continue to be saints, and we can all just get [by with unacceptable working conditions]”.

#### Safeguarding HCPs working in nephrology

The first global wave of the pandemic provided renal HCPs with new and enhanced ways of working. These included successful collaborations between primary and secondary settings (e.g., General Practice and community nursing) and establishing new models of care (e.g., telehealth). However, remote patient care (e.g., virtual clinics) was useful when dealing with reduced capacity but was also described as disjointed and less enjoyable by staff,

“A lot of virtual clinics for us are not terribly helpful because people have to always get their bloods and their urine and their observations and things done, so they have to come and have that done anyway. So when they are here, you might as well see them if they are going to be here anyway. But whenever there was big issues with capacity, then there was the ability to see some people [virtually]…”.

When discussing the future of nephrology, appropriate staffing resources were regarded as key in addressing the impact of COVID-19 on wider health service provision,

“…working on plans to try and clear some of that backlog, but again, staff???… it’s not money or machines or things that are a barrier to that, it’s having enough skilled staff to provide it”.

Numerous supportive COVID-19 initiatives were described by HCPs. These included formal pathways such as a helpline for nursing staff and a counselling service. However, many participants did not avail of these services reporting the most important concerns for staff working during the pandemic were being overlooked by management; for example not being able to gather for breaks was impacting on HCPs well-being. In this study, participants were more likely to avail of informal emotional support from peers or senior colleagues. However, this placed a significant burden on individual staff members. HCPs acknowledged, when the pandemic is over, staff will need ongoing support and this should take the form of the aforementioned supportive COVID-19 initiatives,

"…I think really that senior people within your department are the people who are providing [support] for their own staff, really. And I suppose that then [it] is a burden on them as well as what they are going through personally…"

## Discussion

This is the first study to investigate the experiences of renal HCPs during the first wave of the COVID-19 pandemic, using a multi-methods approach involving an international sample. The results demonstrate that renal HCPs are at high-risk of burnout and mental health distress during the pandemic. Nearly one-third of respondents reported burnout across at least one domain in the current study; highest in emotional exhaustion followed by increased depersonalisation and reduced personal accomplishment. A recent study of HCPs working in intensive care units (ICU) in the United Kingdom (UK) also reported a similar prevalence of emotional exhaustion (38 %; [29]) suggesting that renal HCPs are experiencing similar unprecedented demands in workload intensity as seen in ICU. The present study also found higher mean scores of mental health distress in renal HCPs compared to the UK general population during the same period [30]. Cases of mental health distress in renal HCPs were also higher when compared to HCPs working in emergency medicine (33.3 %), intensive care (41.1 %), and anaesthetics (42.3 %) during the first peak of the COVID-19 pandemic (21/04/2020–05/05/2020; [26]).

Other findings from the current survey describe the level of preparedness, training and supply of PPE as adequate however qualitative interviews report a challenging clinical environment for renal HCPs when caring for a complex patient population during the pandemic. Qualitative insights described the additional clinical burdens brought about by the influx of information and evolving infection control recommendations; from excessive monitoring of PPE to associated communication difficulties affecting patient care. Many obstacles also pertained to redeployment and high levels of staff sickness within renal units. Studies have identified a prospective link between higher burnout and subsequently greater rates of sickness absence which requires further investigation in renal HCPs [31].

Throughout the pandemic there continues to be an urgent need to accelerate protocols to ensure awareness and appropriate implementation of protection in specialisms such as nephrology [32, 33] as well as a growing need for additional and specialist clinical skills in nephrology [34].

Evidence from our qualitative findings also underlined the heightened anxiety, psychological and physical exhaustion experienced by renal HCPs in the early stages of the outbreak, leading to challenges in both their professional and personal lives. International studies have clearly demonstrated an increase in the prevalence of mental health disorders such as depression in HCPs working during the current COVID-19 pandemic [35–37]. Notably, research suggests that this deterioration in mental health will continue to persist even after the pandemic. Roberts et al. [26] reported nearly one-third of HCPs continued to experience psychological distress 30-days post the first COVID-19 global peak. Furthermore, reviews of previous epidemic and pandemic outbreaks in HCPs also demonstrate continued psychological distress up to three years after [38]. It is therefore important to acknowledge the timing of psychological assessments and the importance of follow up to assess the trajectory of psychological distress in HCPs; during and long after a pandemic.

In essence, our qualitative findings also helped to identify the need for appropriate timing and type of support initiatives for renal HCPs. A wide range of support initiatives were described, however many HCPs also highlighted their reliance on informal peer support during the COVID-19 pandemic. This is not a new phenomenon in healthcare, as the most common self-care strategy employed by HCPs to cope with emotional stress appears to be conversing with colleagues who are working together [39]. Organisational peer support programmes which aim to implement a model of peer support using ‘trained peer supporters’ are often used within healthcare [40]. Limited evidence exists on the effectiveness of these programmes however research has shown that a sustained and multipronged campaign is required to increase awareness and trust among staff to participate in such peer support programmes [41].

Improving working conditions is necessary for minimising stress and burnout in clinical staff [42]. COVID-19 has transformed healthcare infrastructure such as the expansion of telehealth and virtual clinics [43]. Renal HCPs described successful collaborations between primary and secondary care however changes to traditional working practices that included non-face-to-face interactions tended to be less appealing to staff. Patients with kidney failure, require precise and personalised care due to several complex management issues relating to comorbidities, medications, and risk of hospitalisation. The future of virtual platforms and technological devices in routine clinical practice is still to be decided, however accounting for HCPs trepidation of the post-pandemic clinical backlog, such interventions (i.e., wearable devices and smartphones) could assist in health management of patients including burnout detection in HCPs [44].

## Conclusions

Work-related stress is likely to be multifaceted; however, few qualitative studies have explored the experiences of HCPs during the COVID-19 pandemic [8]. Qualitative insights from the current study help to demonstrate the serious challenges of managing high-risk patients within renal services during the pandemic. Appropriate resources are paramount to help HCPs feel safe at work and avoid feelings of conflict between family and work. Mental health support during the COVID-19 pandemic would be helpful but how best to protect the well-being and mental health of HCPs requires further study. To date, no published studies have collected data on interventions to improve psychological health and overall well-being for HCPs who face COVID-19 specific challenges [8]. Various interventions have been recommended specifically for frontline HCPs and there is common agreement in the roles of peer and organisation support [26]. Opportunities for psychoeducation (e.g., cognitive behaviour therapy; mindfulness stress reduction) and formal psychological care from mental health professionals (e.g., psychologists or psychiatrists) are limited but should be carefully considered accounting for perceived stigma [45]. This specialist support will be vital during and after the current pandemic [46].

### Limitations

The results of the current study should be interpreted in light of several limitations. The survey sample size was small and conveniently recruited. Responses were predominately skewed towards more senior and experienced nursing staff, and those living in Australia, U.K. and Denmark, creating bias and limiting generalisability to other HCP groups and countries. Increasing the sample to allow for further analysis of different working environments (e.g., outpatients, nephrology ward, home therapy units), professional groups within nephrology within specific countries, and longer follow up (especially as the pandemic continues to be a global problem) would be helpful in identifying factors of burnout and psychological distress. Finally, this study was conducted during the first wave of the global pandemic; subsequent waves have taken place.

### Implications for clinicians, policy and research

Renal HCPs should be considered at high-risk of burnout and psychological distress similar to those working in intensive care and emergency medicine wards during the pandemic. These HCPs require prioritisation for psychosocial support to protect their mental health and well-being if they are to continue to provide high quality complex patient care. New and novel psychoeducational strategies underpinned by psychiatry are required to empower emotional and cognitive skills in HCPs within a re-invigorated wellness culture.

## Supplementary Information

**Additional file 1.**

**Additional file 2.**

## Data Availability

The quantitative datasets analysed during the current study are available from the corresponding author on reasonable request. However, due to the sensitive nature of the qualitative questions asked in this study, respondents were assured raw data would remain confidential and would not be shared.

## Abbreviations

COVID-19: Coronavirus Disease 2019
EDTNA/ERCA: European Dialysis and Transplant Nurses Association/European Renal Care Association
GHQ: General Health Questionnaire 12
HCPs: Healthcare practitioners
MBI: Maslach Burnout Inventory (MBI)
