# Supplementary material for: Experiences of renal healthcare practitioners during the COVID-19 pandemic: a multi-methods approach
Source: BMC Nephrol. 2021 Sep 7;22:301. doi: 10.1186/s12882-021-02500-0 (PMC8421457; doi:10.1186/s12882-021-02500-0)
Supplement: Supplementary file 1 — Additional file 1. [file 12882_2021_2500_MOESM1_ESM.docx]

**Supplementary file 1**

Figure 1. Percentage of burnout by MBI subscales for renal HCPs respondents
