# Supplementary material for: Experiences of renal healthcare practitioners during the COVID-19 pandemic: a multi-methods approach
Source: BMC Nephrol. 2021 Sep 7;22:301. doi: 10.1186/s12882-021-02500-0 (PMC8421457; doi:10.1186/s12882-021-02500-0)
Supplement: Supplementary file 2 — Additional file 2. [file 12882_2021_2500_MOESM2_ESM.docx]

**Supplementary file 2**

**Table 5. Interview questions**

| 1. How did you feel when you first learned of the COVID-19 outbreak in your home country? 2. What do you think are the major (professional or personal) concerns when it comes to coronavirus and patients with renal disease? 3. What were your main concerns relating to the impact of COVID-19? 4. What challenges did you face during the COVID-19 outbreak? 5. What do you feel would help (or would have helped) support your mental health and well-being whilst working as a renal HCP during the COVID-19 outbreak? 6. Thinking to the future, how do you imagine your professional practice will change after COVID-19? 7. Thinking to the future, how do you imagine patient care will change after COVID-19? |
| --- |
